# An Unusual Case of Cerebral Venous Sinus Thrombosis With Negative D-dimer Mimicking Giant Cell Arteritis

**DOI:** 10.7759/cureus.96298

**Published:** 2025-11-07

**Authors:** Ozair Ali, Rahim Abbas, Neha Shaikh, Yaseen Ahmad, Mithun Chakravorty

**Affiliations:** 1 Internal Medicine, Barnsley Hospital NHS Foundation Trust, Barnsley, GBR; 2 Radiology, Glangwili General Hospital, Carmarthen, GBR; 3 Rheumatology, Barnsley Hospital NHS Foundation Trust, Barnsley, GBR

**Keywords:** cerebral venous sinus thrombosis, d-dimer, giant cell arteritis, jaw pain, temporal pain

## Abstract

Cerebral venous sinus thrombosis (CVST) is a rare, potentially life-threatening condition that presents with variable symptoms such as headache, visual disturbance, and seizures, often mimicking an arterial stroke or intracranial lesion. Diagnosis can be challenging, and delays may result in severe outcomes. Although an elevated D-dimer may suggest CVST, a negative result does not exclude it.

We report a rare case of extensive CVST initially treated as giant cell arteritis (GCA) due to unilateral temporal headache, elevated CRP, and a negative D-dimer. A 62-year-old man presented with acute left jaw pain progressing to a left-sided headache. He was initially treated for sinusitis but later developed temporal pain without scalp tenderness or visual disturbance. Referred for suspected GCA, he was started on high-dose prednisolone. CT imaging suggested possible left sigmoid sinus thrombosis, and although his D-dimer was negative, a CT venogram confirmed extensive thrombosis involving the left transverse, sigmoid, and internal jugular veins, with partial extension into the superior sagittal sinus.

Thrombophilia screening revealed a positive lupus anticoagulant, and long-term anticoagulation with warfarin was initiated. This case highlights the need to consider CVST alongside GCA in patients over 50 presenting with temporal headache and jaw pain. A negative D-dimer does not rule out CVST, and a significant thrombus burden may exist despite negative results. Elevated CRP levels can occur in CVST and may be associated with a poorer prognosis, while testing for acquired thrombophilia can help determine the appropriate duration of anticoagulation in unprovoked cases.

## Introduction

Cerebral venous sinus thrombosis (CVST) is a rare but important cause of stroke with a highly variable clinical presentation. Headache is the most frequent manifestation, occurring in up to 90% of cases, and may present in isolation without focal neurological deficits or papilloedema, making diagnosis challenging [1-5]. CVST occurs more commonly in women due to hormonal and pregnancy-associated factors and typically affects younger adults. In the International Study on Cerebral Vein and Dural Sinus Thrombosis (ISCVT), only 8% of the 624 cases occurred in individuals aged 65 years or older [2,6]. Recognised risk factors include thrombophilia (both genetic and acquired), infections such as otitis media and mastoiditis, systemic inflammatory conditions (including systemic lupus erythematosus and inflammatory bowel disease), malignancy, vasculitides such as granulomatosis with polyangiitis, trauma, medications (e.g., steroids), and endocrine disorders [6]. Diagnosis typically requires neuroimaging, such as CT venography (CTV) or MR venography (MRV), and the prompt initiation of anticoagulation is central to management [1].

Giant cell arteritis (GCA) is the most common systemic vasculitis in Western countries, primarily affecting older patients, and remains an important differential diagnosis in older adults presenting with new-onset headache [7]. Being 50 years of age or older is an essential criterion in the 2022 American College of Rheumatology (ACR)/European Alliance of Associations for Rheumatology (EULAR) classification criteria for GCA. On histological examination, GCA demonstrates granulomatous inflammation involving the aortic wall and its major branches, including the extracranial segments of the carotid and vertebral arteries [7]. Common clinical features comprise headache localised to the temporal region, scalp tenderness, abnormal findings on temporal artery examination, and pain or discomfort in the jaw during chewing (jaw claudication) [7]. Usually, blood markers such as ESR or CRP are raised [8]. Complications include permanent loss of vision and therefore require prompt diagnosis and urgent initiation of glucocorticoid treatment to mitigate adverse outcomes.

D-dimer testing is frequently used in the evaluation of suspected venous thromboembolism, but its diagnostic performance in CVST is limited. Although elevated values may support clinical suspicion, a normal D-dimer does not reliably exclude CVST, particularly in patients presenting with isolated headache [1,9-11]. Reported sensitivity varies across studies, highlighting the importance of interpreting D-dimer levels within the appropriate clinical context [12]. Elevated D-dimer levels may also reflect a range of non-thrombotic conditions, including infection, inflammation, malignancy, trauma, and pregnancy [13].

We highlight a case of CVST with a large clot burden in a patient with unusual demographics that mimicked GCA, despite a negative D-dimer.

## Case presentation

A 62-year-old man with a medical history of diet-controlled type 2 diabetes mellitus, hypertension, gastro-oesophageal reflux disease, vitamin B12 deficiency, and hay fever presented to his dentist with a one-day history of persistent left-sided jaw pain. His regular medications included olmesartan, sitagliptin, indapamide, fexofenadine, omeprazole, and oral cyanocobalamin.

A sinus infection was suspected, and he was prescribed amoxicillin and nasal decongestants. Over the following 24 hours, his symptoms worsened and progressed to a left temporo-occipital headache. He reported no visual symptoms, focal neurological deficits, scalp tenderness, or fever. The headache was not exacerbated by standing or lying, and there were no symptoms of meningitis or sinusitis.

He contacted the out-of-hours GP service, where GCA was suspected due to his age and new temporal headache. He was referred urgently to the ED. On arrival, he was afebrile, with normal physical and neurological examination findings. His CRP was mildly elevated at 11 mg/L (reference <5 mg/L), while all other routine blood tests were within normal limits (Table 1). He was started on prednisolone 60 mg daily for suspected GCA and arranged for next-day review in Same Day Emergency Care (SDEC).

**Table 1:** Summary of blood test results. eGFR: Estimated glomerular filtration rate.

Laboratory test (units)	Result (Day 1)	Result (Day 2)	Reference range
Haemoglobin (g/L)	157	159	132-169
White cell count (×10⁹/L)	7.3	7.2	3.7-10.0
Platelet count (×10⁹/L)	156	163	150-450
Serum sodium (mmol/L)	136	134	133-146
Serum potassium (mmol/L)	4.2	4.4	3.5-5.3
Creatinine (µmol/L)	64	76	53-97
eGFR (mL/min/1.73 m²)	>90	>90	>90
Urea (mmol/L)	3.9	5.2	2.5-7.8
CRP (mg/L)	11	11	<5
ESR (mm/hour)	Insufficient sample	5	1-15
D-dimer (µg/mL)	Not performed	<0.50	0.05-0.50

Bloods

The following day (Day 2) at SDEC, symptoms had not improved. Examination remained normal. D-dimer was <0.50 μg/mL. A CT head was obtained due to the atypical headache, revealing hyperdensity in the left sigmoid sinus, which was suspicious for thrombosis. He was admitted urgently and commenced on therapeutic low-molecular-weight heparin (tinzaparin). A thrombophilia screen was performed prior to anticoagulation.

**Figure 1:**
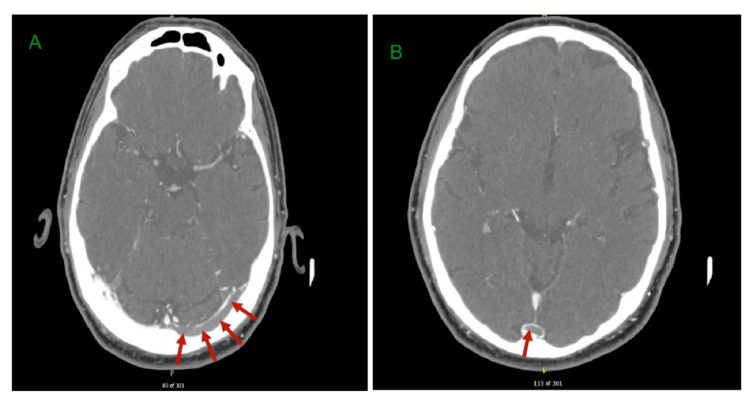
CT venograms. (A) Red arrows indicate a contrast-filling defect in the left transverse sinus, suggestive of thrombosis. (B) Red arrows indicate a contrast-filling defect in the torcula (delta sign).

**Figure 2:**
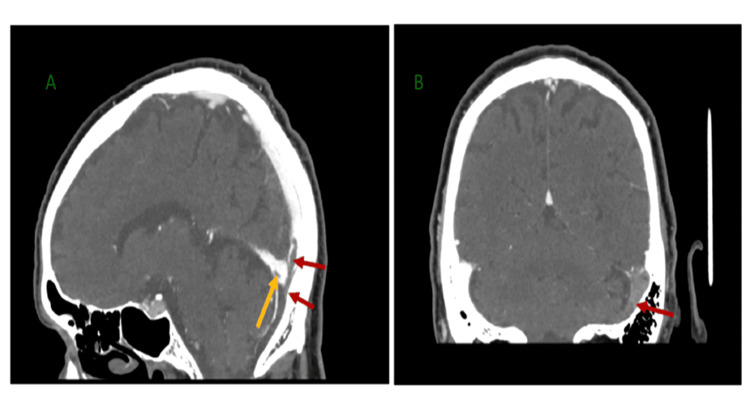
CT venograms. (A) Red arrows indicate a contrast-filling defect/thrombosis in the distal superior sagittal sinus; the yellow arrow indicates a partial filling defect in the straight sinus. (B) Red arrows indicate a contrast-filling defect/thrombosis in the left transverse sinus.

CT venography (CTV) confirmed extensive left-sided venous sinus thrombosis involving the left transverse and sigmoid sinuses, the internal jugular vein, and partial extension towards the superior sagittal sinus from the torcula (Figures 1-2). The cavernous sinus was patent, and no acute intracerebral haemorrhage was seen.

The case was discussed at the neuroradiology multidisciplinary team (MDT) meeting, where neurosurgical intervention was not required, and conservative management with anticoagulation was recommended. ENT and ophthalmology assessments found no contributory pathology or signs of GCA or raised intracranial pressure.

The patient completed a five-day inpatient stay and was discharged symptom-free on therapeutic low-molecular-weight heparin (LMWH).

Initial thrombophilia testing showed a positive lupus anticoagulant, but anti-cardiolipin and anti-β2-glycoprotein-1 antibodies were negative. JAK2 and PNH screens were negative. Due to a family history of unprovoked DVT in a first-degree relative, long-term anticoagulation with warfarin (INR target 2.5-3.5) was initiated after six weeks of LMWH. Repeat lupus anticoagulant testing was arranged; however, at the time of the patient’s next haematology review, the repeat thrombophilia screen was still pending. Two months later, the patient remained clinically well, and his INR was consistently within the therapeutic range.

## Discussion

In this case, GCA was initially suspected rather than CVST, given that the patient was above the age of 50 with a new unilateral headache in the temporal region, jaw pain, and a raised CRP (a CRP level above 10 mg/L meets the 2022 ACR/EULAR criterion for GCA). However, there was a lack of visual symptoms and no scalp tenderness or symptoms of polymyalgia rheumatica. The jaw pain was persistent and not exacerbated by mastication or relieved after chewing, and hence was not suggestive of claudication. The absence of early ESR elevation and the minimal response to initial corticosteroid therapy further supported an alternative diagnosis. Furthermore, the ophthalmology review was unremarkable and did not reveal ocular features of GCA, such as arteritic anterior ischaemic optic neuropathy (AION). No papilloedema was present, which can be seen in patients with CVST. A CT head was requested due to the unusual nature of the headache, and this suggested possible CVST. The D-dimer was negative, but a subsequent CTV confirmed extensive CVST in the same location where the patient’s headache was present, and this was likely to be the sole cause of the headache. All symptoms eventually settled with anticoagulation, and no further glucocorticoids were given. Extracranial internal jugular vein thrombosis may have contributed to his jaw and temporal discomfort, further explaining the clinical presentation. There was no infection specifically in the ear, nose, or throat as determined by the ENT team, and the patient did not develop any symptoms of infection during hospital admission or post-discharge. Therefore, the elevated CRP was likely due to CVST alone and related to thrombotic inflammation, as described in some studies, and may have poorer prognostic implications; however, further research is needed to confirm this [14,15].

We have identified only one previously reported case in which CVST (lateral sinus thrombosis) presented as idiopathic GCA [16]. However, their patient had sixth nerve palsy and ptosis, prompting suspicion of intracranial pathology. Our patient had no focal neurological deficits, which significantly increased diagnostic difficulty.

Negative D-dimer levels can cause diagnostic uncertainty and delay, as this marker is often elevated in CVST cases [17]. Some studies have shown that D-dimer has a high negative predictive value (above 99.8%) for CVST and high sensitivity (95%) in suspected cases [17]. In a study by Kosinski CM et al., 34 out of 35 CVST patients had high (positive) D-dimer levels, giving a negative predictive value of 99.6% and a specificity of 91.2% [17]. The 2017 European Stroke Organisation guideline for the diagnosis and treatment of CVST suggests checking D-dimer before neuroimaging in cases of CVST, except where there is an isolated headache or a long duration of symptoms (more than one week). However, this recommendation was based on observational studies, and the quality of evidence was deemed low [10]. Therefore, further studies are required to enable more robust guidelines in this area.

Up to 10-20% of CVST cases may present with negative or low D-dimer levels, particularly in isolated cortical venous thrombosis [18,19]. Most studies and meta-analyses propose that a negative D-dimer in CVST is likely related to low clot burden and longer symptom duration [17,20]. In our case, despite a very extensive CVST and acute presentation (two days), the D-dimer was negative, which, to our knowledge, has not been reported before. This reinforces the importance of considering CVST even when the D-dimer is negative.

## Conclusions

Patients with acute unilateral headache and jaw pain may present to a variety of clinicians in both primary and secondary care, including GPs, ED, and SDEC. This case highlights the need to consider CVST in older patients (including male patients) with no known risk factors, as CVST can mimic GCA when presenting as a unifocal temporal headache. A negative D-dimer does not exclude CVST, and imaging such as CT or MR venography should still be pursued if clinical suspicion remains high. CRP can be elevated due to CVST alone and may be associated with a poorer prognosis. Early diagnosis and timely initiation of anticoagulation are essential to prevent complications such as venous infarction or haemorrhagic transformation. Acquired thrombophilia screening should be performed in unprovoked cases to help guide the duration and choice of anticoagulation therapy.
